# Immunological Predictors of Nonresponse to Directly Acting Antiviral Therapy in Patients With Chronic Hepatitis C and Decompensated Cirrhosis

**DOI:** 10.1093/ofid/ofx067

**Published:** 2017-04-03

**Authors:** Kate Childs, Elliot Merritt, Aisling Considine, Alberto Sanchez-Fueyo, Kosh Agarwal, Marc Martinez-Llordella, Ivana Carey

**Affiliations:** 1 Liver Sciences, King’s College London, United Kingdom; and; 2 Institute of Liver Studies, King’s College Hospital, London, United Kingdom

**Keywords:** cirrhosis, directly acting antiviral, HCV, hepatitis C

## Abstract

**Background:**

Sustained virological response rates (SVRs) to directly acting antiviral (DAA) therapy for hepatitis C virus (HCV) are lower in decompensated cirrhosis. Markers of innate immunity predict nonresponse to interferon-based HCV treatment; however, whether they are associated with the response to DAAs in patients with decompensation is not known.

**Methods:**

Information on demographics, adherence, viral kinetics, and resistance were gathered prospectively from a cohort with decompensated cirrhosis treated with 12 weeks of DAAs. C-X-C motif chemokine-10 (CXCL-10) level and T-cell and natural killer (NK) cell phenotype were analyzed pretreatment and at 4 and 12 weeks of treatment.

**Results:**

Of 32 patients, 24 of 32 (75%) achieved SVR (responders). Eight of 32 (25%) experienced relapse after the end of treatment (nonresponders). There were no differences in demographics or adherence between groups. Nonresponders had higher CXCL-10; 320 pg/mL (179461) vs 109 pg/mL (88170) in responders (*P* < .001) and differential CXCL-10 dynamics. Nonresponders had lower NK cell frequency, higher expression of activation receptor NKp30, and lower frequency of the NK subset CD56^−^CD16^+^.

**Conclusions:**

Nonresponders to DAAs displayed a different NK phenotype and CXCL-10 profile to responders. Nonresponders did not have poorer adherence or baseline virological resistance, and this shows that immunological parameters are associated with treatment response to interferon-free treatment for HCV in individuals with decompensated cirrhosis.

With the advent of directly acting antiviral (DAA) therapy for hepatitis C virus (HCV), there has been a great increase in the number of patients who can expect to achieve sustained virological response (SVR). In contrast to the historical treatment of pegylated interferon (IFN) and ribavirin, DAAs deliver SVR rates in the order of 90%–95% and higher [1, 2]. Suppression of HCV viral load is almost universal, but a small percentage of patients experience relapse posttreatment. Patients with decompensated cirrhosis have lower SVR rates than those with compensated cirrhosis, with 10%–15% experiencing virological relapse [3–5]. The reasons for this are not clearly defined but may include altered hepatic exposure to DAAs secondary to portosystemic shunting or the existence of viral reservoirs within the fibrotic matrix [6]. Tolerability of ribavirin- containing regimens is also poorer in decompensated disease. Little is known about predictors of failure to achieve SVR with DAAs. Although numerous clinical parameters predicted poor response to pegylated IFN treatment (eg, age, ethnicity, human immunodeficiency virus [HIV] coinfection, insulin resistance, and interleukin [IL]-28b genotype), none of them have been shown to be associated with virological relapse after DAA-based therapy [1, 7–9].

One of the hallmarks of HCV persistence is the failure of both innate and adaptive antiviral immune responses to clear HCV. This results in continual immune activity in the presence of ongoing viremia. Hepatitis C virus ribonucleic acid (RNA) is detected in host cells by Toll-like receptor 3 (TLR-3) or cytosolic RIG-1 helicase-mediated pathways, leading to transcriptional activation of type 1 IFN. Type 1 IFN binds to the cell surface receptor and activates the Jak-STAT pathway, which induces transcription of IFN-stimulated genes (ISGs), which have antiviral activity [10]. During chronic HCV infection, viral replication is sustained despite persistently high ISG expression. Increased type 1 IFNs also activate natural killer (NK) cells, which, in the context of HCV, display a polarized phenotype with increased cytotoxicity, proapoptotic TRAIL production, and decreased cytokine production [11]. CD4 and CD8 T-cell responses are elicited during acute HCV infection but are unable to contain the virus in most individuals; during chronic infection T-cell responses are only detectable at low levels [12]. Persistent antigenic stimulation results in T-cell exhaustion with a sequential loss of antiviral function. The innate immune response contributes to the inadequacy of the adaptive response, because abrogation of IFN signaling in animal models reduces T-cell exhaustion [13]. One hypothesis is that this interaction protects the host from T cell-mediated damage in situations in which virus cannot be removed.

Features of the specific immune response seen in chronic HCV have been associated with a poor response to pegylated IFN [14–16]. Whether they are also associated with the response to DAAs has not yet been ascertained.

The UK Early Access Program was established specifically to treat patients with decompensated cirrhosis with IFN-free DAAs. Compared with other patient populations, this is a group that is more likely to experience treatment failure. Hence, it provides a unique opportunity (1) to clarify whether HCV-induced immune dysfunction influences virological response to DAAs and (2) to identify useful clinical or immunological predictors. In the current study, we prospectively followed a cohort of 32 decompensated cirrhotic patients. We gathered data on patient demographics, clinical parameters, viral factors, and adherence data. Sequential blood cell immunophenotyping and serum cytokine profiling were performed to explore the immunological consequences of HCV treatment and to identify predictors of response to therapy.

## METHODS

### Patient Cohort

We recruited all patient with chronic HCV infection and Child-Pugh B or C cirrhosis who initiated treatment with DAAs at King’s College Hospital as part of the NHS England Early Access Program between August and November 2014. All patients were considered eligible to participate in the current study, provided they had not previously received a liver transplant or were HCV-HIV coinfected. Patients received 12 weeks of sofosbuvir with either daclatasvir or ledipasvir. All patients received weight-based ribavirin. Patients who underwent liver transplantation or died during treatment or experienced major infectious complications were excluded from the final analysis. All patients provided written informed consent. Adherence data was gathered by questionnaire, patient self-report, and pill count at each clinic visit.

Treatment outcome was achieving SVR, which was defined as “response”, and failure to achieve an SVR, which includes virological relapse or breakthrough, was defined as “nonresponse”. In addition to the cirrhotic cohort, we later recruited a smaller cohort of noncirrhotic patients as a comparison group. These were patients who attended between March and June 2015 and were matched to the decompensated cirrhotic cohort by age, genotype, and gender (Supplementary Table).

### Samples

Ethylenediaminetetraacetic acid plasma samples were taken pretreatment (TW0), at treatment week 4 (TW4), and at the end of treatment (TW12) and frozen at −80°C within 2 hours of the blood draw. Sodium heparin samples were taken at the same time point to isolate peripheral blood mononuclear cells (PBMCs) by density gradient centrifugation. The PBMCs were cryopreserved in freezing media at −140°C.

### Hepatitis C Virus Viral Load Measurements

Hepatitis C virus RNA was assayed using the Roche COBAS AmpliPrep/COBAS TaqMan HCV Test, version 2.0. Nonresponders underwent retrospective next-generation sequencing for HCV resistance-associated mutations at baseline and posttreatment with a threshold detection of 10% for NS3, NS5a, and NS5b regions.

### Plasma Cytokine Levels

Measurement of the following cytokines was carried out using the Randox Biochip cytokine array (Randox Laboratories Ltd, Crumlin, London, UK): IL-1 α, IL-1β, IL-2, IL-4, IL-6, IL-8, IL- 10, IL-12, tumor necrosis factor-α, transforming growth factor (TGF)-β, and IL-17α. C-X-C motif chemokine-10 (CXCL-10), IFN-γ, and λ1, λ2, and λ3 were measured using IP-10 Quantikine ELISA, Human IFN-gamma Quantikine ELISA, and Human IL-29/IL-28B (IFN-lambda 1/3) DuoSet ELISA (R&D Systems Europe, Abingdon, UK).

### Blood Immunophenotyping

We carried out immunophenotyping of total PBMC to quantify CD4 and CD8 effector T-cell subsets, T-regulatory (T-reg) cell frequencies, and NK cell frequency and activation receptor profile. Flow cytometry was performed on a BD FACS Canto, and data were analyzed with FlowJo software (TriStar Inc., Ashland, OR) using 3 panels of liquid monoclonal antibodies. All the experiments were performed on frozen PBMCs. To assess T cells, thawed PBMCs were labeled with allophycocyanin (APC) viability dye (Thermo Fisher Scientific, UK), APC Cy7 anti-CD4 (OKT4; BioLegend, London, UK), PerCP anti-CD8 (SK1; BioLegend), PE anti-CCR7 (G043H7; BioLegend), PeCy7 anti-CD45RA (HI100; BioLegend), and fluorescein isothiocyanate (FITC) anti-PD-1 (clone E12.2H7; BioLegend). T-cell subsets were characterized as follows: naive T cells CD45RA^+^CCR7^+^, central memory T cells CD45RA^−^CCR7^+^, effector memory T cells CD45RA^−^CCR7^−^, and terminally differentiated TEMRA effector cells CD45RA^+^CCR7^−^. T-regulatory cells were defined as CD4^+^CD25^high^FoxP3^+^ using FITC viability dye (Thermo Fisher Scientific, Northumberland, UK), APC Cy7 anti-CD4 (OKT4; BioLegend, London, UK), and APC anti-CD25 (2A3; BD Bioscience, Oxford, UK). In addition, intracellular Foxp3 was performed after cell permeabilization (eBioscience, Scotland, UK) using PE anti-FOXP3 (259D; BioLegend). Natural killer T cells (NKT) and NK cells were defined as CD56^+^, CD3 positive and negative, respectively, and were stained with FITC viability dye (Thermo Fisher Scientific), PeCY7 CD3(eBioscience), APC Cy7 anti-CD56 (HCD56; BioLegend), PerCPCy5.5 anti-CD16 (3G8; BioLegend), PE anti-NKp30 (Miltenyi Biotec, Bisley, UK), and APC anti-NKG2D (1D11; BioLegend).

### Statistical Analysis

Continuous variables are expressed as medians (interquartile range). Continuous variables were compared using Mann-Witney *U* test, and discreet variables were compared using Fisher’s exact test. Univariate logistic analysis was carried out with treatment outcome as the dependent variable; variables that were significant in univariate were included in multivariate logistic analysis. *P* values of <.05 were deemed statistically significant. All statistical analysis was carried out using SPSS (IBM SPSS Statistics V22.0).

## RESULTS

### Demographic Factors, Hepatitis C Virus (HCV) Virological Kinetics, HCV Resistance, and Adherence Do Not Differ Between Responders and Nonresponders to Directly Acting Antiviral Therapy

Of 47 patients treated under the Early Access Program within this time period, 7 had exclusion criteria and 8 developed a complication, ie, they received transplants or died before the end of treatment; therefore, 32 patients were included in the analysis. Twenty-four of 32 (75%) patients achieved SVR12 and were defined as responders. Eight of 32 (25%) patients relapsed posttreatment and were defined as nonresponders. No patient experienced virological breakthrough on treatment. All patients were cirrhotic with Child-Pugh score of B7 or greater. The majority of patients were men; ethnicity, age, gender, and model for endstage liver disease (MELD) score did not differ between responders and nonresponders, although there was a trend towards a higher prevalence of genotype 3 (G3) in the nonresponders. The majority of patients were treated with sofosbuvir/ledipasvir including 4 of 6 patients with G3 in the responder group and 5 of 5 patients with G3 in the nonresponder group. Demographics of the participants are shown in Table 1. All patients achieved undetectable HCV RNA by week 10, and 72% of patients achieved undetectable HCV by treatment week 8. There were no differences in baseline or on treatment HCV RNA levels between the responders and nonresponders at any timepoint. Adherence as measured by number of late or missed doses did not differ significantly between responders and nonresponders (Table 2). After next-generation sequencing of the HCV virus in nonresponders at baseline and posttreatment, no patient had baseline resistance; however, 2 of the nonresponders developed mutations in the NS5 region (1 developed Y93H, 1 developed Q30R).

**Table 1.** Baseline Demographic and Liver Disease Characteristics of Responders and Nonresponders to DAA Therapy

Characteristic		**Responders n = 24**	**Nonresponders n = 8**	***P* Value for Comparison**
Age		57 (46, 61)	58 (52, 63)	ns
Male gender		13 (54%)	6 (75%)	ns
Treatment regimen	SOF/LDV	21 (87%)	8 (100%)	ns
	SOF/DCV	3 (13%)	0	
Genotype	1	14 (58%)	2 (25%)	ns
	2	2 (8.5 %)	0	
	3	6 (25%)	5 (63%)	
	4	2 (8.5 %)	1 (12%)	
Genotype 3 vs non-3		6 (25%)	5 (63%)	.08
HCC		3 (12.5%)	3 (37.5%)	.14
MELD score		12.5 (10.7, 14.2)	11 (9.7, 14.4)	.3
Childs-Pugh Score B/C		20 CP B 4 CP C	8 CP B	.5
Previous IFN-based HCV treatment	Treatment naive	12	3	ns
	Toxicity stop	4	2	
	Responder relapser	6	2	
	Nonresponder	2	1	

Abbreviations: CP, Childs-Pugh; DCV, daclatasvir; HCC, hepatocellular carcinoma; HCV, hepatitis C virus; IFN, interferon; LDV, ledipasvir; MELD, model for end-stage liver disease; ns, not significant; SOF, sofosbuvir.

**Table 2.** Virological Characteristics of Responders and Nonresponders to DAA Therapy

Characteristic	**Responders n = 24**	**Nonresponders n = 8**	***P* Value**
Baseline HCV RNA IU/mL	8.8 E^5^ (2.6 E^7^, 1.1 E^6^)	8.8 E^5^ (2.9E^5^, 2.1 E^6^)	.6
TW4 HCV RNA IU/mL	15 (0, 15)	15 (3.7, 19.8)	.3
TW12 HCV RNA IU/mL	Not detected all patients	Not detected all patients	N/A
Number undetectable at TW4	10 (42%)	2 (25%)	.3
Log drop HCV RNA TW0–TW4	4.8 (4.2, 5.4)	4.8 (4.2, 5.6)	.8
Week achieved HCV RNA negative	6 (3.2, 6.7)	6 (4.5, 8)	.2
Number of doses late/missed in 12 weeks	1 (1, 2)	2 (1.5, 3.5)	.1
Baseline resistance associated substitution (RAS)	N/A	Wild-type virus in 8 of 8 patients	N/A
Posttreatment RAS	N/A	1 patient developed Y93H substitution in NS5a, 1 patient developed Q30R substitution in NS5a	N/A

Abbreviations: HCV, hepatitis C virus; N/A, nonapplicable; RAS, resistance-associated substitutions; RNA, ribonucleic acid; TW0, pretreatment; TW4, treatment week 4; TW12, end of treatment.

### Before Initiating Directly Acting Antiviral Therapy, Nonresponders Exhibit Higher CXCL10 Serum Level and Increased Frequency of Circulating NKp30-Positive Natural Killer Cells

Nonresponders had higher median pretreatment CXCL-10 levels; 320 pg/mL (179461) compared with 109 pg/mL (88170) in responders (*P* < .001). To evaluate whether decompensated cirrhotic patients with HCV infection exhibit different CXCL-10 serum levels than those observed in noncirrhotic patients, we recruited an additional cohort of 23 noncirrhotic patients. The median CXCL-10 level in this group was 258 pg/mL (range, 163–368), which was significantly higher than the decompensated responder group (*P* < .001) but not significantly different from the nonresponder group (*P* = .23).

The baseline NK cell phenotype differed between responders and nonresponders. Responders demonstrated a significantly higher NK cell frequency: 7.01% (4.3, 7.9) vs 4.3% (2.9, 5.3) in nonresponders (*P* = .018). Responders also had significantly higher frequencies of the NK subset CD56^−^CD16^+^ (*P* = .004). Total NKG2D expression did not differ between the groups, but total NKp30 expression on NK cells was significantly higher at baseline in nonresponders: 68.4% (range, 45.6–88.7) vs 38.9% (range, 15.8–67.5), *P* = .03 (Figure 3). Responders had significantly higher CD4 TEMRA at baseline compared with nonresponders: 7.7% (range, 3.8–11.4) vs 3.3% (range, 1.9–4.9), *P* = .03. Otherwise, no differences between the phenotype of CD4 and CD8 cells were found (Figure 4).

Of the cytokines assayed at baseline, only TGF-β was significant in univariate analysis, with higher levels associated with nonresponse (*P* = .04). In univariate analysis, with treatment outcome as the dependent variable, baseline CXCL-10, TGF-β, and total NKp30 and NK cell frequency were significant at the 0.05 level. In the final logistic regression model of baseline variables, only CXCL-10 remained significant with a *P* value of .02, the model predicted 95% of responders to DAA therapy.

### The Dynamics of CXCL-10 Serum Levels During Directly Acting Antiviral Treatment Differ Between Responders and Nonresponders

**Figure 1.**
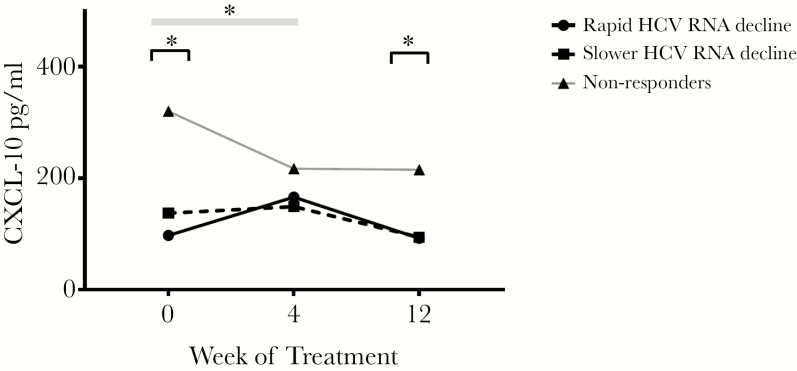
C-X-C motif chemokine-10 ([CXCL-10] pg/mL) change over time by groups: rapid decline in hepatitis C virus (HCV) ribonucleic acid (RNA) (undetectable by week 6 and achieved sustained virological response rate [SVR]), slower HCV RNA decline (undetectable after week 6 and achieved SVR), nonresponder (did not achieve SVR). The * signifies *P* < .05, the black line shows comparison between groups at a time point, and the gray box shows difference in fold change between groups over time.

CXCL-10 was significantly higher in nonresponders than in responders both at baseline and at the end of treatment: 215 pg/mL (115378) compared with 93 pg/mL (44166) (*P* = .041) in responders. However, all of the nonresponders experienced a fall in CXCL-10 from baseline to TW4, and responders showed an increase in CXCL-10 from baseline to TW4 (*P* = .002 for difference in CXCL-10 fold change). Among responders, those who achieved undetectable HCV RNA by week 6 of treatment had a significantly greater fold increase in CXCL-10 at TW4 compared with those who only suppressed HCV RNA at TW6 or later (*P* = .04) (Figure 1). Nonresponse to DAA therapy is associated with lower NK cell frequency, elevated NK NKp30 expression, and an increase in CD56^−^CD16^+^ NK cells during treatment.

Natural killer cell frequency was higher at baseline and TW4 in responders; however, total NKp30 expression on NK cells was higher at baseline, on treatment, and at the end of treatment in nonresponders. No changes over the course of treatment in either responders or nonresponders were noted (Figure 3).

**Figure 2.**
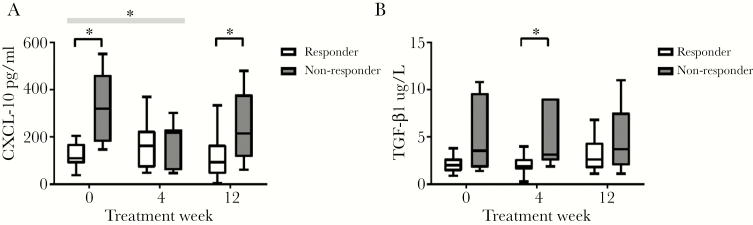
C-X-C motif chemokine-10 ([CXCL-10] pg/mL) and transforming growth factor (TGF)-β (µg/L) change over time in responders and nonresponders. The * signifies *P* < .05, the black line shows comparison between groups at a time point, and the gray box shows difference in fold change between groups over time.

**Figure 3.**
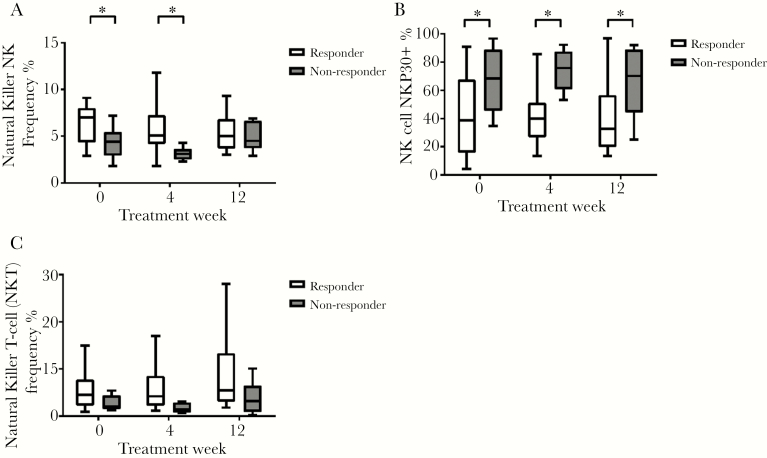
Natural killer (NK) cell frequency, NKp30-positive NK cells, and NK T-cells (NKT) frequency change over time in responders and nonresponders. The * signifies *P* < .05, the black line shows comparison between groups at a time point, and the gray box shows difference in fold change between groups over time.

**Figure 4.**
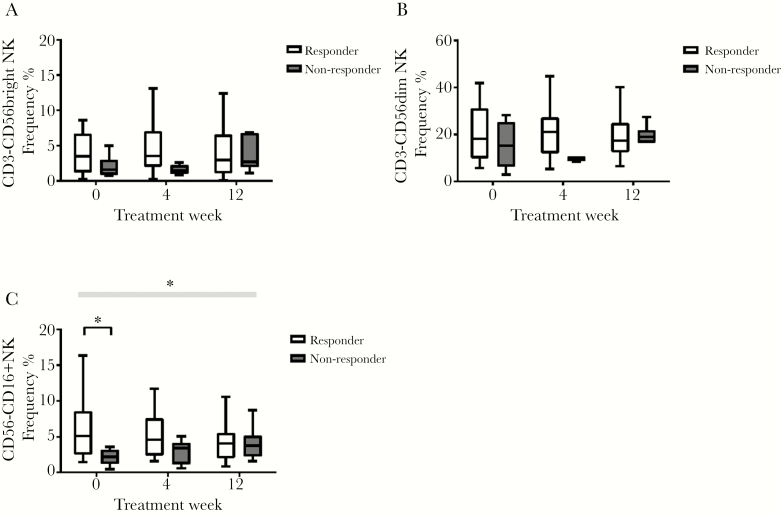
CD56^bright^ natural killer (NK), CD56^dim^ NK, CD56^−^CD16^+^ NK frequency change over time in responders and nonresponders. The * signifies *P* < .05, the black line shows comparison between groups at a time point, and the gray box shows difference in fold change between groups over time.

Baseline frequencies of the NK cell subset CD56^−^CD16^+^ were significantly higher in responders 5.1% (range, 2.5–8.5) than nonresponders 2.1% (range, 1.2–3.1) (*P* = .004). The frequency of the CD56^−^CD16^+^ population decreased over the course of treatment in responders but increased in nonresponders so that by the end of treatment, numbers were similar: 4.0% (2.5, 8.5) in responders and 3.8% (2.2, 5.1) in nonresponders (Figure 4). The fold change from pretreatment to end of treatment was significantly different between responders and nonresponders (*P* value for fold change = .001).

The TGF-β was significantly higher at TW4 in nonresponders: 3.2 µg/L (range, 2.5–9.1) compared with 1.8 µg/L (range, 1.5–2.6) in responders (*P* = .02) (Figure 2). There was no difference in levels of IFN-γ, λ1, λ2, and λ3 or other cytokines at baseline or on treatment.

**Figure 5.**
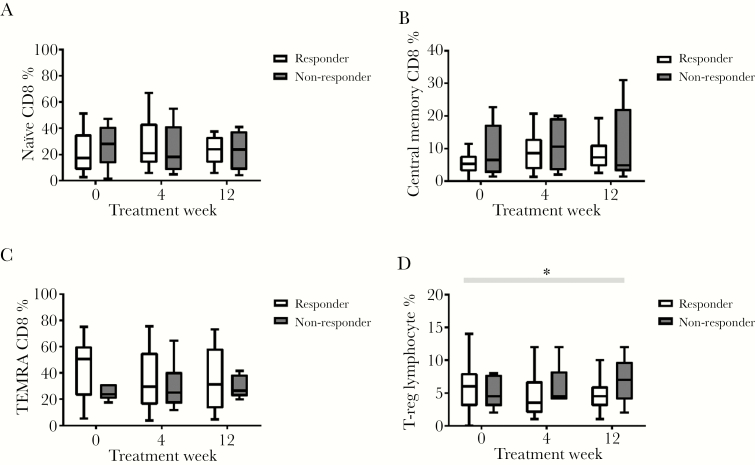
Naive CD8, central memory CD8, TEMRA CD8, and T-regulatory (T-reg) cell frequency change over time in responders and nonresponders. The * signifies *P* < .05, the black line shows comparison between groups at a time point, and the gray box shows difference in fold change between groups over time.

Responders and nonresponders exhibited similar frequencies of CD4 and CD8 effector T-cell subsets throughout the 3 timepoints (Figure 5). In contrast, T-reg cell frequencies differed between the 2 groups: although there was no difference at baseline, T-reg frequency decreased in responders and increased in nonresponders during treatment (*P* = .02 for difference in fold change) (Figure 5).

## DISCUSSION

There are few studies that have described the characteristics of individuals who fail to respond to DAAs, and the majority of these have focused on HCV viral resistance [17, 18]. We show that in patients with decompensated cirrhosis, nonresponders to DAA therapy did not have baseline HCV resistance mutations nor poorer adherence than responders. However, the data reported here indicate that there is an association between the response to DAA and a number of immunological parameters. CXCL-10 serum levels and NK immunophenotype were particularly informative.

 CXCL-10 is a chemokine known to be released from HCV-infected livers as a result of increased ISG expression. Serum CXCL-10 is highly correlated with intrahepatic CXCL-10 mRNA expression [14] and is often used as a surrogate marker of hepatic ISG expression [19]. Thus, increased serum CXCL-10 and elevated pretreatment ISG expression are both predictors of nonresponse to pegylated IFN-based treatment [20, 21].

We found that in patients with decompensated cirrhosis, elevated baseline CXCL-10 was associated with nonresponse to DAAs. There are few data on CXCL-10 levels in the context of decompensation because these patients are often excluded from studies [22]. In our study, the majority of patients with decompensation responded to DAAs, and this group had significantly lower CXCL-10 levels than a noncirrhotic comparison group. The lower CXCL-10 levels in decompensation may reflect the reduced number of hepatocytes; lower HCV RNA and transaminase levels are also seen in decompensated disease [23]. However, the nonresponder patients had similar CXCL-10 levels to the noncirrhotic group, in whom SVR rates exceed 95%.

Directly acting antiviral treatment resulted in a decrease in CXCL-10 in all nonresponders but an increase in CXCL-10 levels in responders, with the fastest virological responders showing the greatest increase. Our findings that elevated CXCL-10 at baseline is associated with nonresponse, but upregulation of CXCL-10 on treatment is associated with response is directly analogous to the well reported mechanism of ISG expression as a predictor of response to pegylated IFN [14]. That IFN signaling still plays a role even in IFN-free treatment fits with hepatic gene expression data from Meissner et al [24], who showed that hepatic IFN-α expression increased during successful IFN-free treatment for HCV. At the end of 12 weeks of treatment, a cross-sectional comparison between responders and nonresponders showed that hepatic ISG expression was higher in responders [24]. These authors suggest an ongoing role for IFN signaling even during DAA therapy for HCV.

Our finding that CXCL-10 increased in responders on DAA treatment differs from the results of Meissner et al [25] and Spaan et al [25] who found a consistent decline in CXCL-10 in all patients as HCV RNA declined. A potential explanation for our dissimilar findings is that all of our patients had decompensated cirrhosis, whereas in the other studies most patients were noncirrhotic, and none were decompensated. Two pathways of hepatic CXCL-10 induction are known: (1) a direct signaling pathway where recognition of HCV RNA by pattern recognition receptors triggers transcription within the hepatocyte; and (2) indirect production in response to type I, II, and III IFNs produced by Kupffer cells, stellate cells, and sinusoidal epithelial cells (nonparenchymal cells [NPCs]) [26]. In decompensated cirrhosis, with a reduced hepatocyte mass and elevated IFN-γ levels [27], CXCL-10 production may be primarily driven by the production of IFNs by NPC. We also saw a significant elevation in CXCL-10 level at the end of treatment in nonresponders. This may represent an ongoing innate immune response to low-level residual HCV viremia before overt virological relapse.

The parallel with the relationship linking overactivation of the innate immune system with nonresponse to pegylated IFN extends to our findings in NK cells. We found that nonresponders demonstrated a different NK cell profile compared with responders, with proportions of NK cells expressing the natural cytotoxicity receptor NKp30 higher in nonresponders at all timepoints. NKp30 is an activation receptor that is upregulated in response to IFN-α [28, 29], and high NKp30 expression before IFN-based therapy predicts nonresponse to treatment and is analogous to upregulated ISG expression as a marker of overactivation of the innate immune system [30]. We also saw a decline in T-reg cells over the course of treatment in responders but an increase in nonresponders. T-regulatory cells are suppressed by IFN, and their activity favors HCV chronicity, so upregulation of IFN signaling during treatment in responders could explain the decline in T-reg cells seen in this group [31].

That responders had a higher frequency of the CD56^−^CD16^+^ NK cells subset was an unexpected finding. The CD56^−^CD16^+^ subset of NK cells is enriched in patients with HIV and HCV infection and is a dysfunctional subset with impaired cytotoxicity and cytokine production and a loss of polyfunctionality likened to exhaustion seen in T cells [32]. This subset declined over the course of treatment in responders while increasing in nonresponders.

We considered other causes for the difference in response between the groups. We first considered the number of patients with G3 infection. G3, which has been most challenging in the DAA era, was slightly overrepresented in the nonresponder group, although this was not statistically significant. Most of the patients with G3 were treated with sofosbuvir/ledipasvir and ribavirin, which is not a currently recommended treatment, but it was the only option available at the time of the study. It seems unlikely that this was the main reason for nonresponse because the majority of G3 patients in the responder group also received this combination. We also considered whether the higher proportion of patients with G3 in the nonresponders could explain the immunological differences observed. Although CXCL-10 levels are lower in genotype 3 HCV than G1 [20], G3 does upregulate IFN signaling in NPCs to a greater extent than G1 HCV [33]. Because we hypothesize that in decompensated cirrhosis the source of CXCL-10 is NPCs, this could partially explain the higher levels seen in the nonresponders.

Another factor to consider is the relatively higher number of hepatocellular carcinoma (HCC) cases in the nonresponder group. This difference was also not significant, although with small group numbers type 2 errors may occur. The presence of HCC may play a role, because HCC has recently been revealed as a risk factor for failing DAA therapy [34]. This could either be because HCC serves as a sanctuary for virus, or it could be because the immune deficits that predispose to HCC also predispose to nonresponse to DAAs. An immune evasion technique of HCC is to increase myeloid-derived suppressor cells that inhibit NK via NKp30; elevated NK NKp30 expression in HCC is also associated with worse prognosis [35, 36].

Limitations of our study include the small number of subjects, although because our patients had decompensated cirrhosis, we report a relatively high number of nonresponders. The differences that we describe between responders and nonresponders may not be generalizable to the general population of noncirrhotic patients with HCV. We were also confined to investigating the peripheral immune response rather than the hepatic response.

## CONCLUSIONS

With well tolerated DAAs, response rates in patients with decompensated cirrhosis remain lower than in those without cirrhosis. This study shows that responders and nonresponders to DAAs differ in their immunological characteristics and not all nonresponse is a function of poor adherence. Although nonresponders may subsequently achieve an SVR with longer course of treatment [37], failing 12 weeks of treatment is undesirable because there is a risk of further decompensation and death. Baseline CXCL-10 is a simple test that could predict response to therapy in the majority of patients with decompensated cirrhosis and allow an extended duration of treatment in patients with higher levels.

In summary, we show that in patients with decompensated cirrhosis, nonresponders to DAA therapy displayed differences in CXCL-10 profile and NK phenotype. This supports an ongoing role for the innate immune system even in IFN-free treatment for HCV.

## Supplementary Data

Supplementary materials are available at *Open Forum Infectious Diseases* online. Consisting of data provided by the authors to benefit the reader, the posted materials are not copyedited and are the sole responsibility of the authors, so questions or comments should be addressed to the corresponding author.

## Supplementary Material

ofx067_suppl_Supplementary_S1_data

ofx067_suppl_Supplementary_S2_data

ofx067_suppl_Supplementary_S3_data

ofx067_suppl_Supplementary_Table1
