# Supplementary figures and images for: Immunological Predictors of Nonresponse to Directly Acting Antiviral Therapy in Patients With Chronic Hepatitis C and Decompensated Cirrhosis
Source: Open Forum Infect Dis. 2017 Apr 3;4(2):ofx067. doi: 10.1093/ofid/ofx067 (PMC5450903; doi:10.1093/ofid/ofx067)

## Slide 1
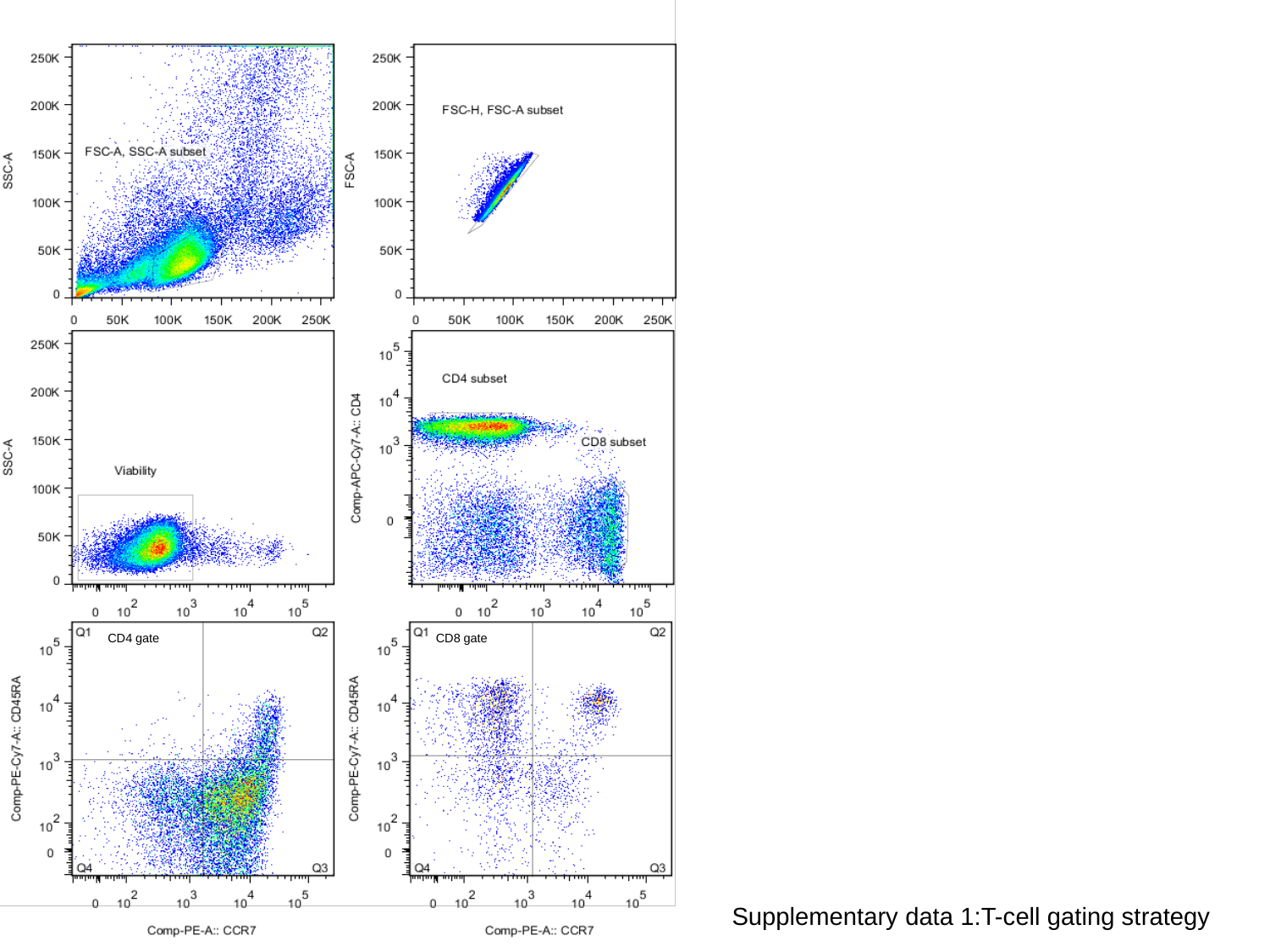

CD4 gate
CD8 gate
Supplementary data 1:T-cell gating strategy

Supplement: ofx067_suppl_Supplementary_S1_data [file ofx067_suppl_supplementary_s1_data.pptx]

## Slide 1
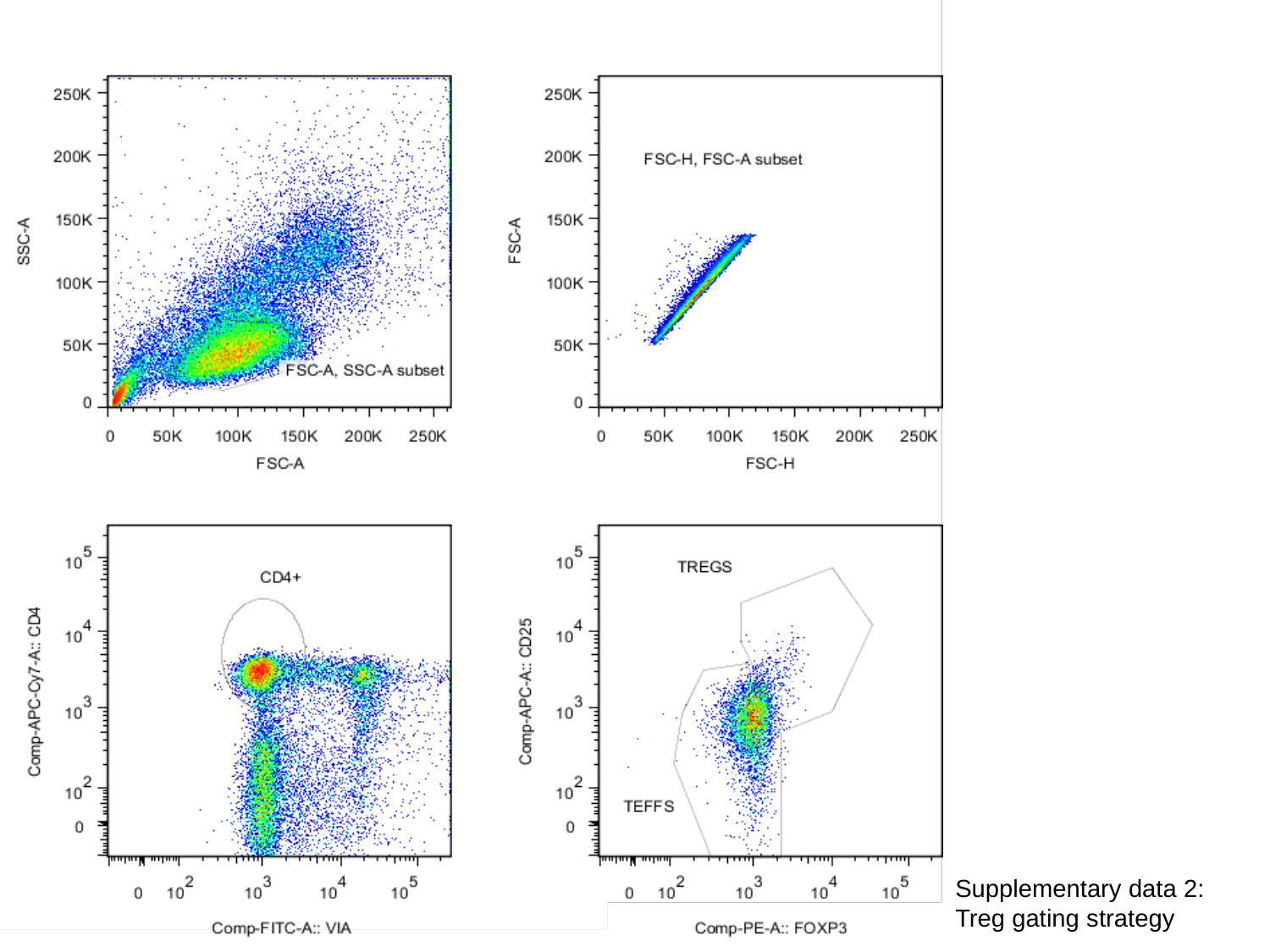

Supplementary data 2: Treg gating strategy

Supplement: ofx067_suppl_Supplementary_S2_data [file ofx067_suppl_supplementary_s2_data.pptx]

## Slide 1
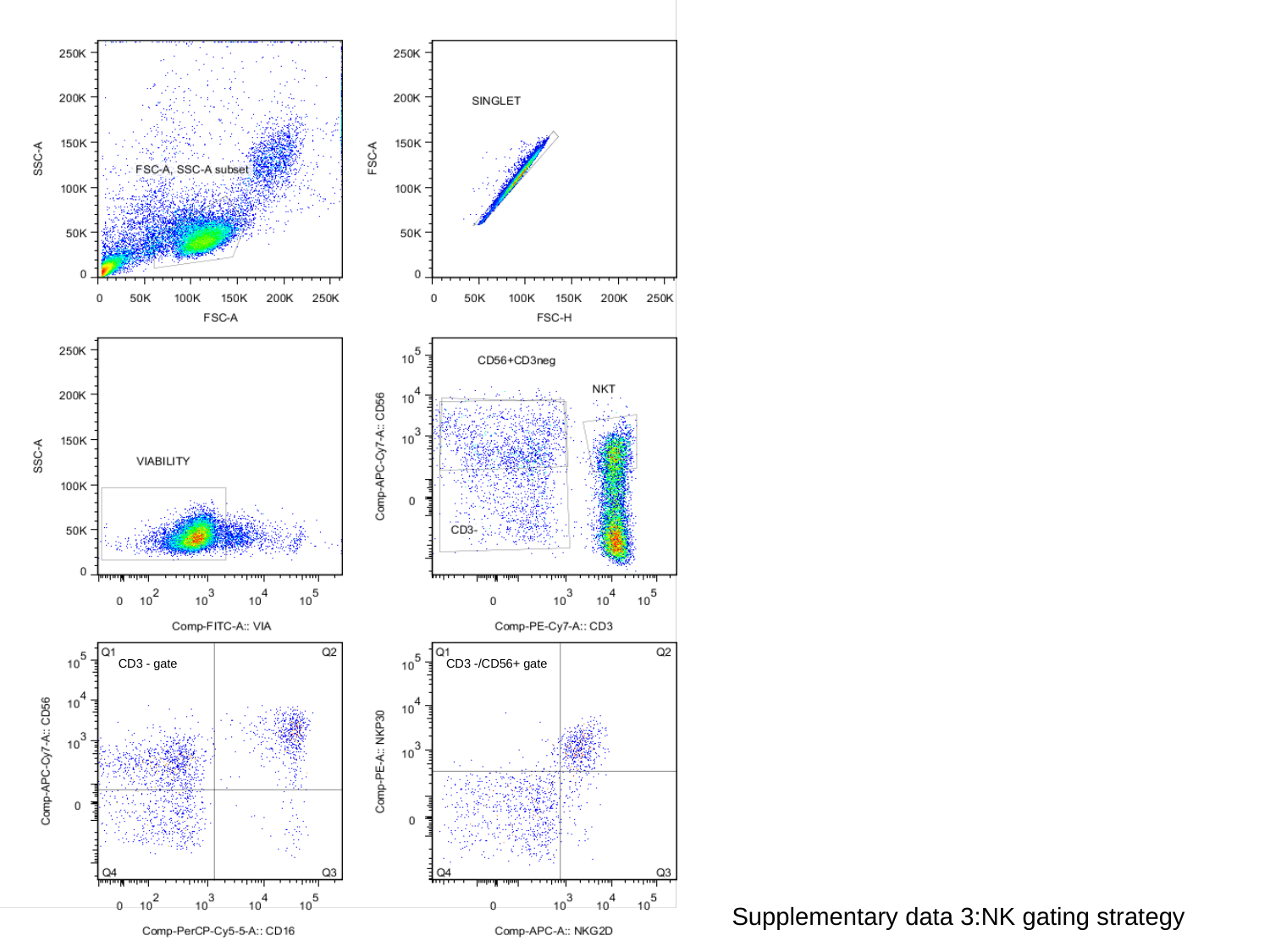

CD3 - gate
CD3 -/CD56+ gate
Supplementary data 3:NK gating strategy

Supplement: ofx067_suppl_Supplementary_S3_data [file ofx067_suppl_supplementary_s3_data.pptx]
