# Supplementary material for: Immunological Predictors of Nonresponse to Directly Acting Antiviral Therapy in Patients With Chronic Hepatitis C and Decompensated Cirrhosis
Source: Open Forum Infect Dis. 2017 Apr 3;4(2):ofx067. doi: 10.1093/ofid/ofx067 (PMC5450903; doi:10.1093/ofid/ofx067)
Supplement: ofx067_suppl_Supplementary_Table1 [file ofx067_suppl_supplementary_table1.docx]

| **Supplementary Table 1**  **Comparison group of non-cirrhotic patient** | | **Non-cirrhotic**  **n=25** |
| --- | --- | --- |
| Age | | 51 (42, 54) |
| Ethnicity | Caucasian | 18 |
|  | Black | 3 |
|  | Asian | 2 |
|  | Mixed | 2 |
| Male gender | | 17 |
| Genotype | 1 | 18 |
|  | 2 |  |
|  | 3 | 5 |
|  | 4 | 2 |
| Genotype 3 vs non-3 | | 20% |
| Fibroscan score (kPa) | | 5.3 (4.6, 5.6) |
| HCC | | none |
